# An atlas for human brain myelin content throughout the adult life span

**DOI:** 10.1038/s41598-020-79540-3

**Published:** 2021-01-11

**Authors:** Adam V. Dvorak, Taylor Swift-LaPointe, Irene M. Vavasour, Lisa Eunyoung Lee, Shawna Abel, Bretta Russell-Schulz, Carina Graf, Anika Wurl, Hanwen Liu, Cornelia Laule, David K. B. Li, Anthony Traboulsee, Roger Tam, Lara A. Boyd, Alex L. MacKay, Shannon H. Kolind

**Affiliations:** 1 Physics and Astronomy, University of British Columbia, Vancouver, BC Canada; 2 International Collaboration on Repair Discoveries (ICORD), University of British Columbia, Vancouver, BC Canada; 3 Radiology, University of British Columbia, Vancouver, BC Canada; 4 Medicine (Neurology), University of British Columbia, Vancouver, BC Canada; 5 Pathology and Laboratory Medicine, University of British Columbia, Vancouver, BC Canada; 6 Biomedical Engineering, University of British Columbia, Vancouver, BC Canada; 7 Department of Physical Therapy, University of British Columbia, Vancouver, BC Canada

**Keywords:** Ageing, Magnetic resonance imaging, Myelin biology and repair, Neural ageing, Brain

## Abstract

Myelin water imaging is a quantitative neuroimaging technique that provides the myelin water fraction (MWF), a metric highly specific to myelin content, and the intra-/extra-cellular T_2_ (IET2), which is related to water and iron content. We coupled high-resolution data from 100 adults with gold-standard methodology to create an optimized anatomical brain template and accompanying MWF and IET2 atlases. We then used the MWF atlas to characterize how myelin content relates to demographic factors. In most brain regions, myelin content followed a quadratic pattern of increase during the third decade of life, plateau at a maximum around the fifth decade, then decrease during later decades. The ranking of mean myelin content between brain regions remained consistent across age groups. These openly available normative atlases can facilitate evaluation of myelin imaging results on an individual basis and elucidate the distribution of myelin content between brain regions and in the context of aging.

Myelin water imaging (MWI) is a quantitative magnetic resonance imaging (MRI) technique that uses a multi-echo T_2_ relaxation sequence to characterize myelin^1^. T_2_ relaxation in normal human brain generally has three main components: a short T_2_ component originating from myelin water (T_2_ < 40 ms), an intermediate T_2_ component attributed to intra- and extra-cellular water (40 ms < T_2_ < 200 ms), and a long T_2_ component representing cerebrospinal fluid (CSF) (T_2_ >> 200 ms)^2^. Healthy corticospinal tracts and white matter (WM) affected by pathology can also exhibit an additional component with 200 ms < T_2_ < 800 ms^3^. Myelin water fraction (MWF), the fraction of signal contribution from myelin water, has been histopathologically validated as a biomarker for myelin^4^, and has demonstrated significant variation in myelination between different brain structures not often apparent with other MRI techniques^5^. The geometric mean T_2_ of the intra- and extra-cellular water compartment (IET2) is sensitive to tissue water and iron, with lower values expected for lower water content and higher iron concentration^6,7^.

To identify changes or abnormalities in T_2_ relaxation metrics in the context of development, aging or disease, it is crucial to have an accurate picture of these metrics in the healthy population. One method to characterize normal brain T_2_ measures is to analyse MRI data using a representative reference for anatomical images (a “template”) with a corresponding representative quantitative metric map (an “atlas”). Combining datasets reduces noise from individual measurements and from biological variation between subjects, which can arise from age, sex, ethnicity, pathology, hemispheric asymmetry, or other factors. If demographic factors can sufficiently characterize biological variations, then atlases could be used to evaluate quantitative MRI metrics on an individual basis^8^. Voxel-wise analysis of individual MWF maps has been used to study brain and spinal cord in a variety of diseases, but with relatively small sample sizes and limited characterization of possible demographic factor influences^9,10^.

Atlas-based methods have been used to probe the relationship between MWF and demographic factors in healthy adult brain but with conflicting results. Opposing positive^11^ and negative^12^ linear correlations between age and MWF have been reported, while other research found relatively few brain regions where any age-MWF correlation existed^13^. Furthermore, some studies have suggested that an inverted U-shaped quadratic model better describes the age-MWF relationship^14,15^, contrary to literature where linear models performed best^12^.

The need for normative MWI data and the inconsistent findings of how MWF changes with age motivated the current study. We acquired high-resolution data with the reference multi-echo T_2_ relaxation method for MWI in a large subject group with a broad age range, and used gold-standard atlas creation methodology to:

1. Create an optimized anatomical template with accompanying MWF and IET2 atlases, tissue segmentations, and regions of interest (ROIs).
2. Investigate the strength of age and sex as explanatory variables for inter-subject MWF and IET2 variations.
3. Determine the extent to which ranking of MWF and IET2 between brain regions is consistent across age groups.

## Results

**Figure 1:**
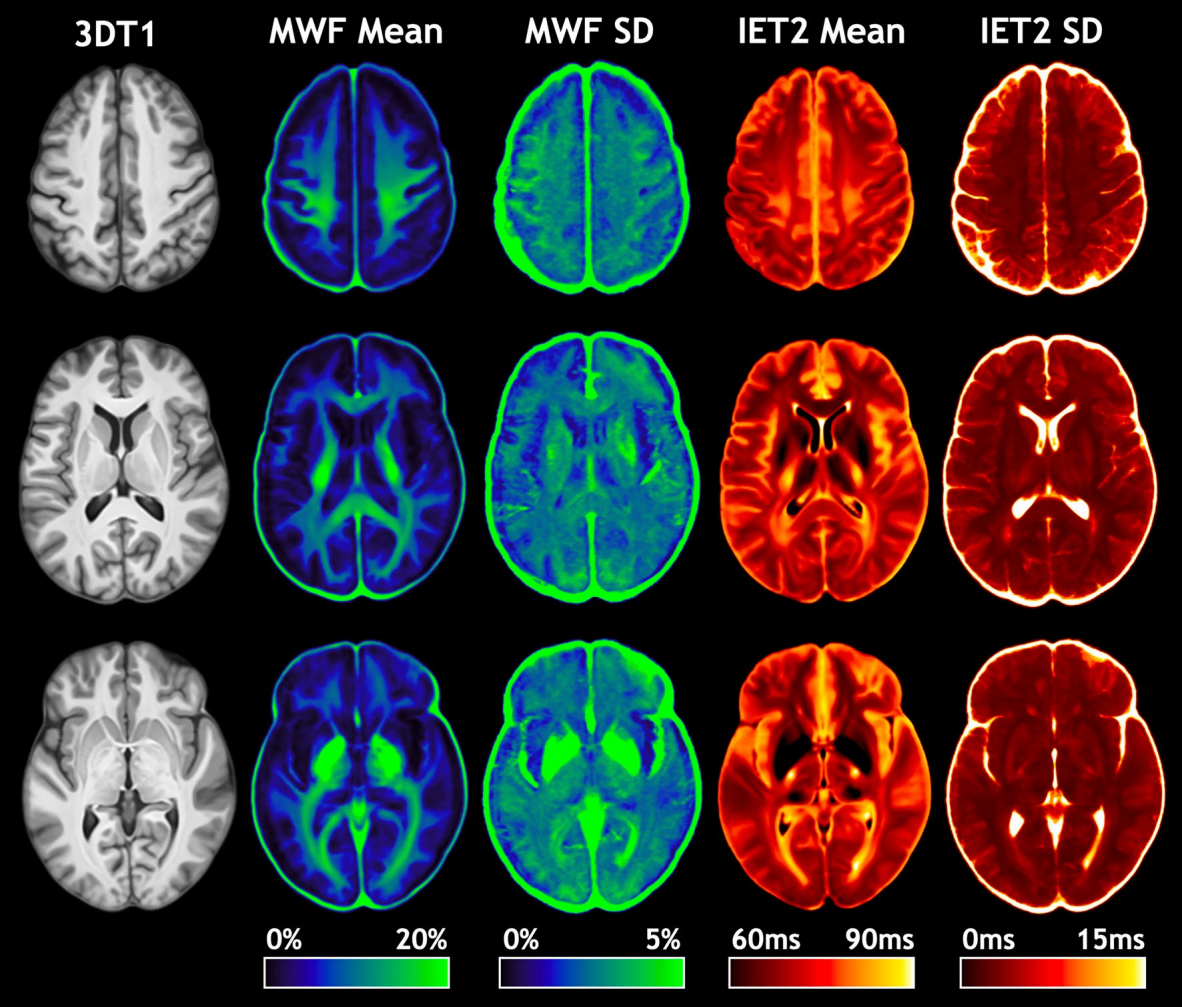
Representative slices of the T_1_-weighted anatomical (3DT1) template, myelin water fraction (MWF) mean and standard deviation (SD) atlases, and geometric mean T_2_ of intra- and extra-cellular water (IET2) mean and SD atlases.

Due to incidental findings, MWF and IET2 maps from two subjects were excluded from atlas creation and subsequent analysis. Representative slices of the T_1_-weighted anatomical (3DT1) template and mean and standard deviation (SD) atlases for MWF and IET2 are shown in Fig. 1.

### Linear regression with age

Correlation coefficients between age and mean MWF and IET2 in all ROIs are presented in Table 1. MWF correlated significantly with age in the corpus callosum (body *r* = 0.388 *P* < 0.001, splenium *r* = 0.337 *P* < 0.001), internal capsule (anterior *r* = 0.359 *P* < 0.001, posterior *r* = 0.369 *P* < 0.001, retrolenticular *r* = 0.306 *P* < 0.01), external capsules (*r* = 0.335 *P* < 0.001), fornix (*r* = 0.358 *P* < 0.001), all WM ROIs combined (*r* = 0.289 *P* < 0.01), WM mask (*r* = 0.326 *P* < 0.01), and gray matter (GM) mask (*r* = 0.403 *P* < 0.001).

**Table 1:** Pearson correlation coefficients and *P*-values for correlation between age and mean myelin water fraction (MWF), and age and mean geometric mean T_2_ of intra- and extra-cellular water (IET2), for each region of interest. Statistically significant correlations are **bold** for *P* < 0.01 and bold* for *P* < 0.001.

Region of Interest	MWF		IET2	
	*r*-value	*P*-value	*r*-value	*P*-value
Genu of corpus callosum	0.071	0.488	**0.623**	7.15E−12*
Body of corpus callosum	**0.388**	7.95E−05*	**0.437**	6.73E−06*
Splenium of corpus callosum	**0.337**	6.96E−04*	**0.290**	**3.82E−03**
Cerebral peduncle	**0.360**	2.70E−04*	**− 0.364**	2.27E−04*
Anterior limb of internal capsule	**0.359**	2.87E−04*	− 0.025	0.808
Posterior limb of internal capsule	**0.369**	1.86E−04*	0.234	0.020
Retrolenticular part of internal capsule	**0.306**	**2.21E−03**	0.111	0.277
Anterior corona radiata	0.214	0.035	**0.548**	5.16E−09*
Superior corona radiata	0.226	0.026	**0.479**	5.90E−07*
Posterior corona radiata	0.221	0.029	**0.331**	8.62E−04*
Posterior thalamic radiation	− 0.080	0.435	**0.478**	6.54E−07*
Sagittal stratum	0.115	0.258	**0.328**	9.62E−04*
External capsules	**0.335**	7.36E−04*	0.082	0.420
Cingulum (cingulate gyrus)	0.207	0.041	0.209	0.039
Fornix (cres) / Stria terminalis	**0.358**	2.92E−04*	0.133	0.193
Superior longitudinal fasciculus	0.198	0.051	**0.469**	1.13E−06*
All white matter ROIs	**0.289**	**3.91E−03**	**0.424**	1.33E−05*
White matter mask	**0.326**	**1.07E−03**	0.200	0.0481
Gray matter mask	**0.403**	3.91E−05*	**− 0.658**	1.83E−13*

IET2 correlated significantly with age in corpus callosum (genu *r* = 0.623 *P* < 0.001, body *r* = 0.437 *P* < 0.001, splenium *r* = 0.290 *P* < 0.01), corona radiata (all *P* < 0.001), posterior thalamic radiation (*r* = 0.478 *P* < 0.001), sagittal stratum (*r* = 0.328 *P* < 0.001), superior longitudinal fasciculus (*r* = 0.469 *P* < 0.001), all WM ROIs combined (*r* = 0.424 *P* < 0.001), and GM mask (*r* = − 0.658 *P* < 0.001).

**Figure 2:**
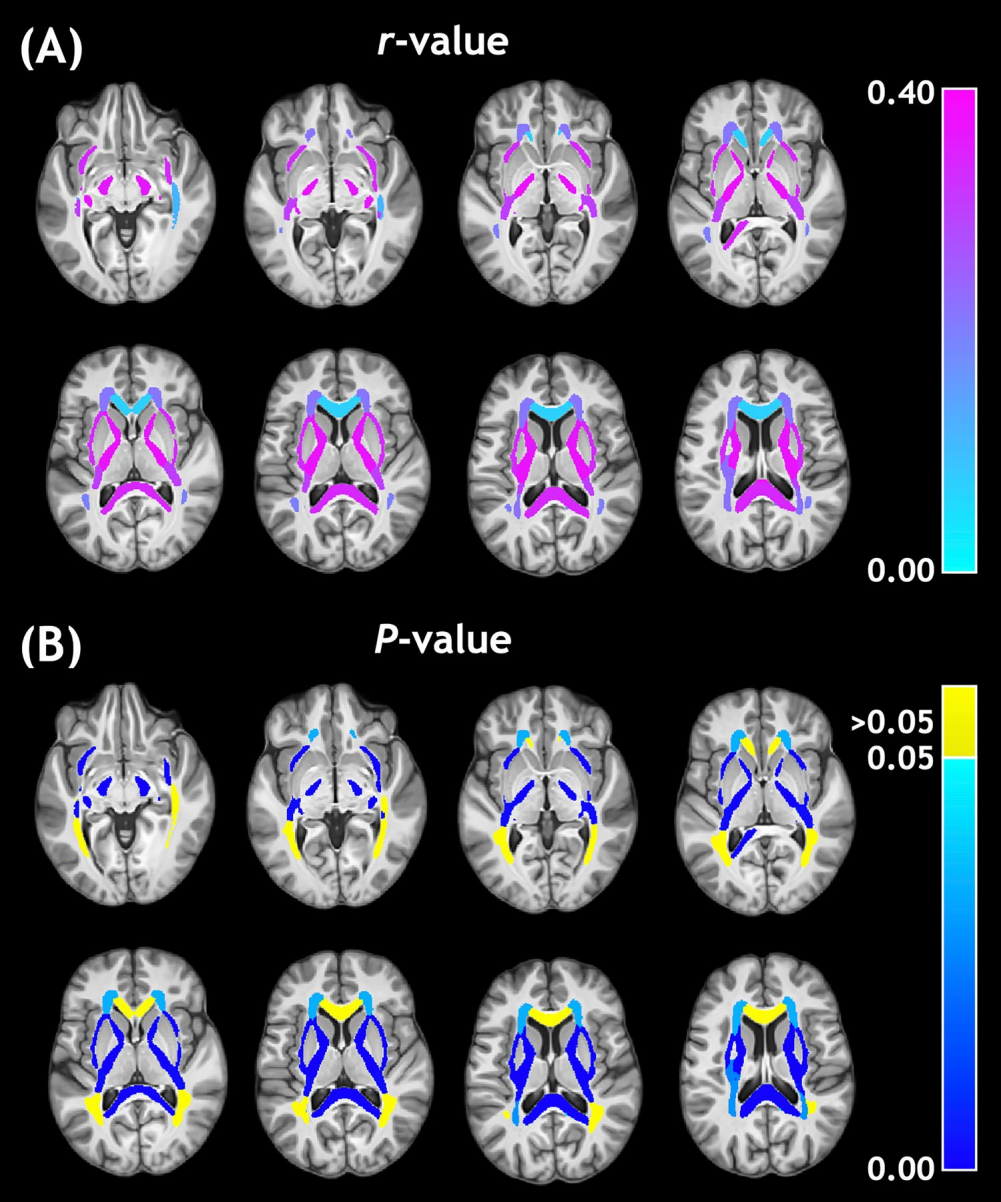
Pearson (**A**) correlation coefficients (*r*-values) and (**B**) *P*-values for the relationship between age and myelin water fraction.

**Figure 3:**
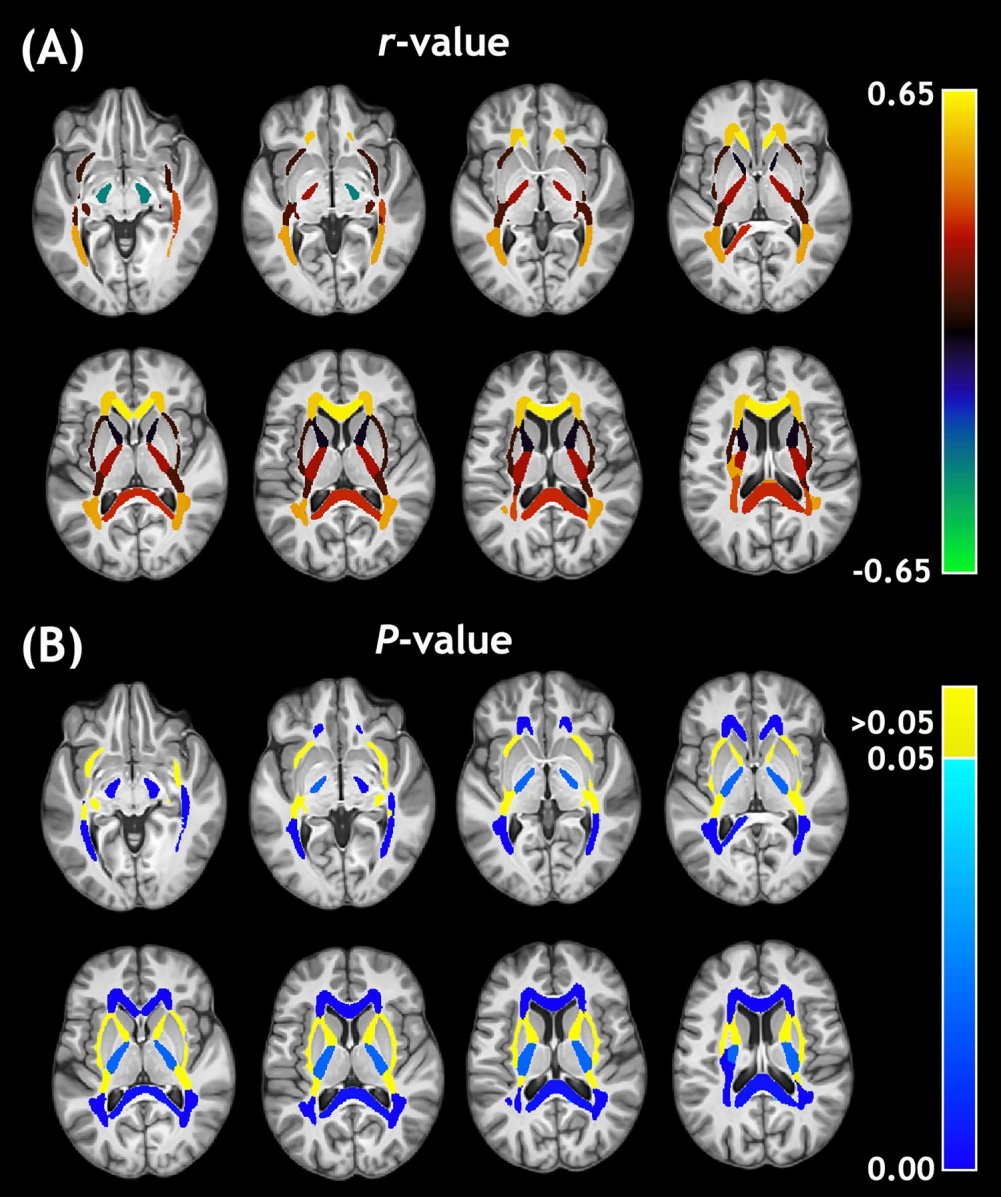
Pearson (**A**) correlation coefficients (*r*-values) and (**B**) *P*-values for the relationship between age and geometric mean T_2_ of intra- and extra-cellular water.

For visual assessment, these correlation values were assigned to their respective ROI masks and presented in Fig. 2 for MWF and Fig. 3 for IET2. Large ROIs composed of multiple smaller regions were omitted from Figs. 2 and 3 so that the smaller ROIs were not obscured. The strongest, highest significance age-MWF correlations in Fig. 2 were clustered towards central or central-posterior brain regions. The age-IET2 correlations in Fig. 3 showed the opposite spatial pattern, with higher significance age-IET2 correlations in peripheral brain regions and stronger correlation coefficients generally favouring anterior brain regions.

### Multiple linear regression including Age^2^, sex

Multiple linear regression adjusted r-values as well as *P*-values for the age and age^2^ model terms are presented in Table 2, for mean MWF and IET2 values in all ROIs. Some ROIs without significant Pearson correlations reached significance for multiple regression with the quadratic age term, for MWF: superior and posterior corona radiata (both *P* < 0.01), sagittal stratum (*P* < 0.01), cingulum (*P* < 0.001), and superior longitudinal fasciculus (*P* < 0.001) and for IET2: internal capsule (anterior and posterior *P* < 0.001, retrolenticular *P* < 0.01), external capsules (*P* < 0.001), cingulum (*P* < 0.01), and WM mask (*P* < 0.001). Multiple regression with the additional age^2^ term provided a higher r-value (adjusted) than linear correlation for MWF in all ROIs and for IET2 in all ROIs except the splenium of the corpus callosum. In multiple linear regression with age and sex, the binarized sex variable was not significant for MWF (*P* > 0.1) or IET2 (*P* > 0.01) correlations in any ROI, which indicated that there were no significant differences in the age-MWF or age-IET2 relationships between sexes.

**Table 2:** Coefficients and *P*-values for multiple linear regression between a quadratic polynomial in age and mean myelin water fraction (MWF), and between a quadratic polynomial in age and mean geometric mean T_2_ of intra- and extra-cellular water (IET2), for each region of interest.

Region of Interest	MWF			IET2		
	Adjusted *r*-value	*P*-value (Age)	*P*-Value (Age^2^)	Adjusted *r*-value	*P*-value (Age)	*P*-Value (Age^2^)
Genu of corpus callosum	0.184	0.024	0.029	0.640	0.317	0.022
Body of corpus callosum	0.408	0.010	0.051	0.453	0.263	0.056
Splenium of corpus callosum	0.346	0.032	0.106	0.263	0.819	0.475
Cerebral peduncle	**0.467**	6.14E−05*	5.19E−04*	**0.394**	**7.17E−03**	0.035
Anterior limb of internal capsule	**0.459**	7.78E−05*	6.54E−04*	**0.486**	1.92E−07*	1.69E−07*
Posterior limb of internal capsule	**0.463**	1.08E−04*	9.11E−04*	**0.421**	5.16E−04*	1.01E−04*
Retrolenticular part of internal capsule	**0.371**	**2.30E−03**	**9.59E−03**	**0.298**	**3.87E−03**	**1.94E−03**
Anterior corona radiata	**0.327**	**1.54E−03**	0.041	**0.628**	**3.81E−03**	7.44E−05*
Superior corona radiata	**0.348**	7.99E−04*	**2.38E−03**	**0.548**	0.015	8.84E−04*
Posterior corona radiata	**0.323**	**2.12E−03**	**5.75E−03**	**0.402**	0.030	**5.81E−03**
Posterior thalamic radiation	0.163	0.068	0.048	**0.530**	0.040	**3.22E−03**
Sagittal stratum	**0.258**	**4.62E−03**	**7.20E−03**	0.351	0.185	0.058
External capsules	**0.423**	3.69E−04*	**2.21E−03**	**0.423**	1.75E−05*	7.74E−06*
Cingulum (cingulate gyrus)	**0.369**	2.21E−04*	6.35E−04*	**0.318**	0.015	**5.08E−03**
Fornix (cres) / Stria terminalis	**0.441**	3.00E−04*	**2.12E−03**	0.178	0.112	0.068
Superior longitudinal fasciculus	**0.338**	7.31E−04*	**1.88E−03**	0.475	0.438	0.103
All white matter ROIs	**0.389**	5.34E−04*	**2.34E−03**	**0.499**	0.015	**1.34E−03**
White matter mask	**0.464**	2.09E−05*	1.53E−04*	**0.401**	5.19E−04*	1.28E−04*
Gray matter mask	**0.448**	**1.49E−03**	0.011	**0.755**	1.01E−10*	1.09E−07*

Statistically significant linear (age) and quadratic (age^2^) contributions to the regression model are **bold** for *P* < 0.01 and bold* for *P* < 0.001.

### ROI value rank across age groups

**Figure 4:**
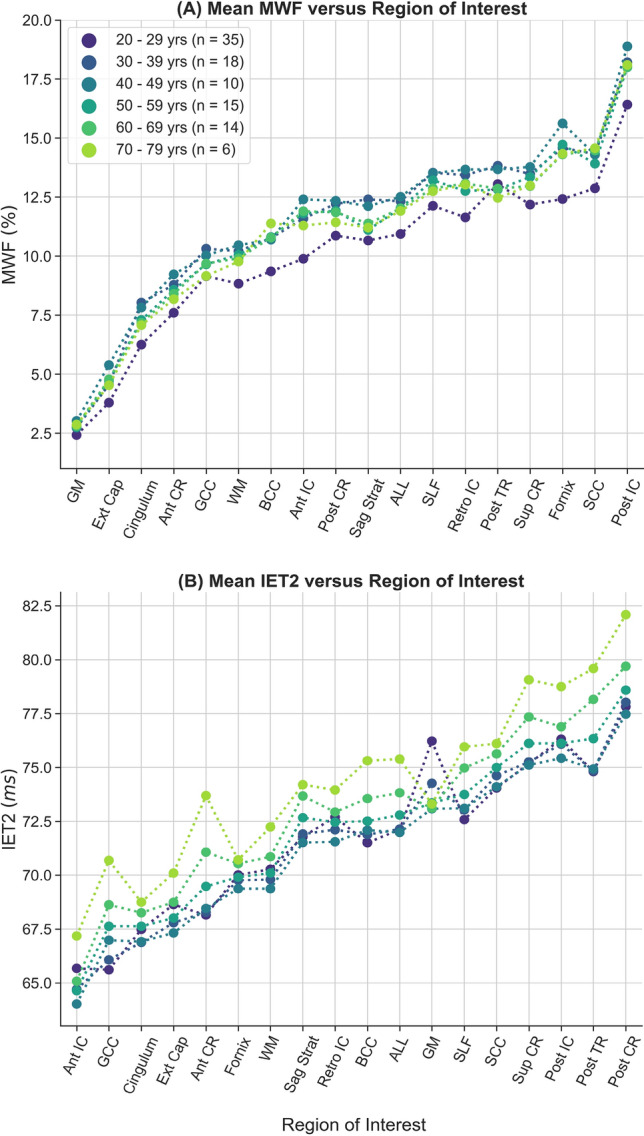
Rank of average A) myelin water fraction (MWF) and B) geometric mean T_2_ of intra- and extra-cellular water (IET2) for subjects grouped by decade of age within gray/white matter (GM/WM), external capsules (Ext Cap), cingulum, anterior/posterior/superior corona radiata (Ant/Post/Sup CR), anterior/posterior/retrolenticular internal capsule (Ant/Post/Retro IC), sagittal stratum (Sag Strat), all JHU labels (ALL), superior longitudinal fasciculus (SLF), posterior thalamic radiation (Post TR), fornix (Fornix), and genu/body/splenium of corpus callosum (G/B/S CC).

Mean metric ROI values for all subjects, grouped by decade of age, were plotted in Fig. 4A for MWF and Fig. 4B for IET2 to demonstrate the ranking of metric values between ROIs and across different age groups.

**Figure 5:**
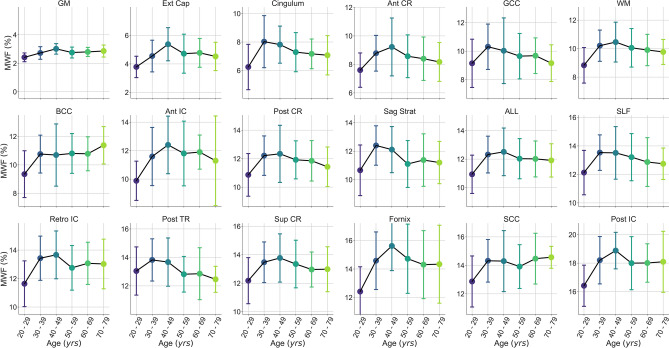
Mean myelin water fraction (MWF) values versus subject age (grouped by decade) within gray/white matter (GM/WM), external capsules (Ext Cap), cingulum, anterior/posterior/superior corona radiata (Ant/Post/Sup CR), anterior/posterior/retrolenticular internal capsule (Ant/Post/Retro IC), sagittal stratum (Sag Strat), all JHU labels (ALL), superior longitudinal fasciculus (SLF), posterior thalamic radiation (Post TR), fornix (Fornix), and genu/body/splenium of corpus callosum (G/B/S CC). Uncertainty bars were calculated as the standard deviation of values across subjects within each age group.

**Figure 6:**
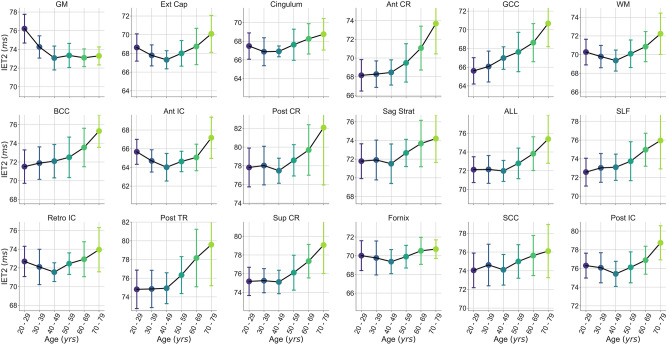
Mean geometric mean T_2_ of intra- and extra-cellular water (IET2) values versus subject age (grouped by decade) within gray/white matter (GM/WM), external capsules (Ext Cap), cingulum, anterior/posterior/superior corona radiata (Ant/Post/Sup CR), anterior/posterior/retrolenticular internal capsule (Ant/Post/Retro IC), sagittal stratum (Sag Strat), all JHU labels (ALL), superior longitudinal fasciculus (SLF), posterior thalamic radiation (Post TR), fornix (Fornix), and genu/body/splenium of corpus callosum (G/B/S CC). Uncertainty bars were calculated as the standard deviation of values across subjects within each age group.

To provide a visual representation of the age-MWF and age-IET2 relationships, mean metric values were plotted versus subject age (grouped by decade) for all ROIs in Fig. 5 for MWF and Fig. 6 for IET2. In most ROIs, MWF showed an inverted U-shaped trend of increasing then decreasing MWF with age, while IET2 values generally followed the opposite pattern.

## Discussion

The template, atlases, tissue segmentations, and ROIs have been made openly available^16^ with example code for their creation^17^. The template was previously used for a MWI repeatability study^18^, and the template and atlases are being used for analyses of multiple studies at our institution.

The 3DT1 template shows excellent anatomical detail, indicative of accurate inter-subject alignment of 3DT1 images during template creation. This is demonstrated by sharp WM/GM delineation within regions with fine-scale structure, such as the cerebellum, where blurring or mixed contrast would indicate limited performance of registrations. The posterior protrusion of the left occipital pole, characteristic of the brains of right-handed individuals, is expected for a representative template of predominantly right-handed subjects^19^. This characteristic structure is also reflected in the mean MWF and IET2 atlas patterns, which suggests good intra-subject alignment between the 3DT1 anatomical images and gradient and spin-echo (GRASE) MWI images.

Atlas SD maps generally did not show higher-intensity borders outlining brain structures, which would indicate misalignment. With measurement noise reduced by the large number of datasets, high SD within anatomical structures is expected to originate from biological variation between subjects.

Although brain extraction generally improved template quality during our testing, inaccuracies drastically reduced registration quality. We found that intensity normalization, voxel-wise squaring to accentuate GRASE echo 1 image contrast, constraining similarity metric calculation to brain regions, and careful choice of reference template (based on image contrast, resolution, and field-of-view) facilitated consistent and accurate brain extractions.

Image registration implicitly requires that the same features are present in both inputs. High degree-of-freedom registration techniques, such as the symmetric diffeomorphic normalization transformation used here, are especially susceptible to mismatched input image features caused by brain extraction errors, pathology, or other factors. The assumptions of image registrations break down in brain regions without inter-subject correspondence, such as within cortical folds, or in those with fundamental differences in structure, such as duplication of Heschl’s gyrus^20^. Misregistration in regions such as these would contribute to hyperintensity in the atlas SD maps, possibly visible at the furthest anterior and posterior edges of the brain.

Diffeomorphic transformations, such as those used to create our template, intrinsically preserve the topology of input images^21^. Therefore, local intra-structure relationships present in quantitative maps, but not necessarily present on anatomical MRI contrast, are preserved during inter-subject alignment of large-scale MR-visible structures. Considering previous work which has suggested that the cyto-architectural layout of the brain is generally consistent^22^, topology-preservation is an essential characteristic for research attempting to bridge the gap between MRI macroanatomy and histological microanatomy. Although subtle quantitative metric map features could be explicitly aligned using registrations, this would introduce a circularity bias whereby the statistical significance of future comparisons to the atlas is maximized^23^. Furthermore, this methodology would cause naturally occurring variation between individuals to be artificially minimized, which is antithetical to the purpose of an atlas^23^.

Positive correlations between age and mean MWF (Table 1, Fig. 2) are consistent with the results of Flynn et al., who found positive linear correlations between MWF and age with 27 subjects (mean age 36.5 years) acquiring a single 10 mm thick slice of 2D MWI data^11^. Billiet et al. (n = 59, age range 17–70 years) used a 3D gradient and spin-echo (GRASE) sequence to acquire high-resolution full-brain T_2_ relaxation MWI data in under 12 min, but found relatively few regions with positive correlations between MWF and age^13^. Our results disagree with those of Faizy et al. (n = 45, median age 36 years), which showed strong negative correlations between MWF and age using data from two different 3D GRASE sequences, acquired with lower 3 and 5 mm isotropic resolution^12^. However, relatively low-resolution data (27 mm^3^ and 125 mm^3^ versus our 10 mm^3^ MWI voxel volume) could have influenced their correlations by decreasing MWF values for older subjects with brain atrophy, due to partial-volume effects with CSF.

Our significant multiple linear regressions with a quadratic age term, especially strong for some ROIs (Table 2), are supported by the inverted U-shape relationship between age and mean MWF shown in Fig. 5. These results agree with those reported by Arshad et al., who investigated six brain regions (n = 61, 18–84 years) and found a quadratic increase then decrease in MWF with age^14^. Papadaki et al. found a similar quadratic relationship in a larger subject group (n = 90, 22–81 years) but were limited to coarse spatial analysis of MWF in each brain lobe, due to acquiring five 2D MWI slices with 8 mm thickness and an 8 mm inter-slice gap^15^. Various limitations, such as limited brain coverage, low resolution, limited age range, and modest sample size, make it difficult to draw clear conclusions from the conflicting results previously reported for the age-MWF relationship. However, our results suggest that MWF does in fact follow a rapid increase during the third decade of life, followed by a non-linear trend where myelin content plateaus then decreases in later decades. Our observed trend in MWF agrees with literature suggesting that myelination continues until the sixth decade of life^24^ and is corroborated by the parabolic relationship found with age for previous studies using alternative quantitative MRI measures sensitive to myelin^25,26^. The location of maximum MWF appears to differ somewhat between brain regions in Fig. 5. However, we avoided assigning an age of peak MWF in each ROI because the exact value of the quadratic peak would be “substantially affected by seemingly irrelevant factors, such as the age-range sampled”^27^ and therefore biased by the specific characteristics of our subject group.

The myelin-age relationship has also been studied using an alternative approach to MWI known as multicomponent-driven equilibrium single-component observation of T_1_ and T_2_ with Bayesian Monte Carlo analysis (BMC-mcDESPOT)^28^, which uses a more efficient steady state acquisition to provide a surrogate measure for the MWF^29^. Using a large sample and wide age range, Bouhrara et al. (n = 106, range 22–94 years) found a quadratic association between myelin values and age^28^, similar to that found by Arshad et al. and Papadaki et al.^14,15^. Although Bayesian Monte Carlo analysis improves the stability of mcDESPOT, which has been shown to provide biased myelin estimates with unpredictable fluctuations^30^, BMC-mcDESPOT values remain overestimated compared to the reference multi-echo T_2_ relaxation method^31^ and have not been validated with comparison to histological staining. Furthermore, BMC-mcDESPOT estimates of MWF show little contrast between white matter regions, for example the genu and splenium of the corpus callosum^28,29^, which is inconsistent with T_2_ relaxation MWF results^14^ and is not reflective of myelin content variations reported by post mortem studies^32^.

Compared to the BMC-mcDESPOT results of Bouhrara et al., our MWF in WM ROIs shows much more variation between individual subject values^28^. This could reflect the increased myelin-specificity of our 3D multi-echo T_2_ relaxation MWI method, which is less sensitive to magnetisation transfer or other effects that could explain the unusually high, homogeneous MWF provided by mcDESPOT and its derivative techniques^28^. Our results also show increased variation in the trajectory of MWF with age between regions, which may better reflect the heterochronous brain development trajectories expected from literature^33^.

Mean IET2 increased with age in most WM ROIs (Table 1, Fig. 3), possibly due to increasing water content with age, and decreased in GM, likely related to increasing iron concentration with age^34^. Differences in axon diameter, which would affect the rate of exchange between the myelin and intra- and extra-cellular water compartments, likely also play a significant role in driving IET2 regional differences^35^. Our findings indicate that controlling for age can account for a significant amount of inter-subject MWF and IET2 variations.

Excluding subjects aged < 25 years (26 subjects) eliminated significance of both Pearson correlations and multiple linear regression for MWF (*P* > 0.05, except for posterior thalamic radiation *P* < 0.01) but did not drastically change IET2 results. Although this suggests that the age-MWF relationship is driven by subjects < 25 years of age, it could also be related to having relatively few subjects > 70 years of age and none > 78 years of age. Although some ROIs show a roughly inverse pattern between MWF in Fig. 5 and IET2 in Fig. 6, different dependence of the age-MWF and age-IET2 relationships on subjects < 25 years of age confirms that MWF and IET2 provide unique, complementary information.

The addition of a binarized sex variable did not significantly affect the age-MWF (*P* > 0.1) or age-IET2 (*P* > 0.01) relationship. Faizy et al. also reported the absence of sex-related differences for MWF in any WM ROIs^12^. Although distinct developmental patterns have been reported between sexes during early childhood^36^ and for young adults with median age < 25 years^37^, our results suggest that male and female distributions of myelin content converge at adulthood. Our results emphasize the importance of controlling for age, but not necessarily sex, when comparing MWF and IET2 values between adults.

Mean MWF and IET2 both demonstrate clear ranking between brain structures for different age groups (Fig. 4), indicating that relative metric values are generally consistent for adults. Offsets between ranking of subjects grouped by age further support the strength of the age-MWF and age-IET2 relationships. However, this relationship may not hold in children or adolescent brains, which develop with a spatially and temporally non-uniform pattern^36,38,39^.

A number of limitations should be considered with respect to our study. The presence of iron can artificially increase MWF values by decreasing T_2_ relaxation times^7^. Although most iron in WM is found in oligodendrocytes, co-localized with myelin, iron content is an important consideration in processes where iron and myelin changes occur independently, such as aging^34^. This highlights the value in characterizing healthy brain MWF and IET2 values across a wide range of ages, as provided by our atlas. Future study could combine MWI data with diffusion models to correct some of the effect of orientation on T_2_ relaxation values, and subsequently MWF and IET2 values^40^. Kumar et al. have developed an analysis approach using 3D spatial correlations to improve robustness to the spatial and temporal noise in MWI data^41^. Although the resulting maps are both aesthetically and quantitatively improved, the prohibitive analysis time was not practical for use in this study, at least until computational time can be reduced using GPU acceleration or other approaches.

Our atlases can be supplemented with additional datasets by processing images, registering to the template, and re-calculating the atlases using additional quantitative maps. Age-specific atlases can be generated by simply re-calculating atlases using a subset of the available data, from subjects within an appropriate age range. Although the subject group presented here has a relatively even spread of subjects between the third to eighth decade of life, future studies could benefit from additional subjects over 70 years of age and the inclusion of datasets from children and adolescents. Standardized collection of more demographic information, such as years of education, could prove similarly beneficial to developing expectations for myelin in the brain.

With enough data, a spatiotemporal “4D atlas” could be used to generate a continuous spectrum of age-specific atlases. Similarly, longitudinal data could better characterize MWF evolution across time by removing noise from inter-subject biological variability.

Previous studies have used quantitative MRI to generate maps related to human brain myelo-architecture^42^, usually by combining multiple imaging modalities. In vivo myelo-architecture maps could potentially be generated from MWI data by modelling the rate of exchange between the myelin water and intra- and extra-cellular water compartments, which is thought to be related to the underlying axon radius and myelin sheath thickness^35^. Accounting for exchange could also facilitate more robust myelin quantification in regions with especially fast or slow exchange rates.

Atlases could be aligned with atlases of spatially localized gene expression information^43,44^, even in tandem with temporal information. This could accelerate synergistic investigations that combine MWI metrics with genetics, which have already proven valuable by demonstrating an association between hemispheric asymmetries and genetic variation in *PLP1*^45^.

In conclusion, our template, atlases, tissue segmentations, and ROIs provide an optimal framework for using MWI to study healthy myelin development or demyelinating disease. Age, but not sex, can be used to better characterize expected values for healthy adult MWF and IET2. MWF generally increases during the third decade of life, plateaus around the fifth decade, then decreases in later decades. Lastly, we showed that the ranking of MWF and IET2 values between brain structures remains consistent across different adult age groups.

## Methods

### MRI

Data was collected retrospectively from studies performed at the UBC MRI Research Centre on a 3.0 T Achieva (Philips Healthcare, Best, The Netherlands) using an 8-channel head coil. Each study was approved by the University of British Columbia Clinical Research Ethics Board and subjects provided written informed consent. All methods were performed in accordance with the relevant guidelines and regulations.

Inclusion criteria required that subjects had no history of brain disease or injury and that datasets included a high-resolution, sagittal T_1_-weighted MPRAGE sequence (3DT1) and a specific 48 echo gradient and spin-echo (GRASE) MWI sequence, with scan parameter criteria as follows.

3DT1 images had 0.8 or 1.0 mm isotropic resolution and field-of-view (FOV) = 256x256x160 mm^3^ or larger. Although 3DT1 MPRAGE sequence parameters varied slightly between studies, most were acquired with readout repetition time (TR) / echo time (TE) = 8.1/2.5 ms, inversion time (TI) = 1052 ms, shot interval = 3000 ms, acquisition time = 6 min 26 s. MWI data were acquired using a 3D multi-echo GRASE sequence with TR = 1073 ms, TE = 8 ms, ΔTE = 8 ms, 48 echoes, slice oversampling factor = 1.3, FOV = 230x190x100 mm^3^, acquired resolution = 1x2x5 mm^3^ (20 slices), reconstructed resolution = 1 × 1x2.5 mm^3^ (40 slices), sensitivity encoding acceleration factor of 2, and acquisition time = 7 min 31 s^46^.

Data from 100 healthy subjects were included in our study. MRI data were collected in PAR/REC format to ensure that all conversion, processing, and analysis was identical. For subjects with data available from multiple visits, only the first was included.

Age and sex demographic information was collected for all subjects (median age 38 years, range 20—78 years, 58F/42 M). Handedness information was available for 72 subjects but was not diverse enough for meaningful investigation (70 right-handed, 2 left-handed). The subject group varied in age from the third to the eighth decade of life (number of subjects: 35, 19, 11, 15, 14, and 6, respectively) and was well spread across the fourth to seventh decades.

### MWI analysis

T_2_ distributions were produced from GRASE data in native space, before any transformations were applied, using a temporally-regularized non-negative least-squares fitting algorithm with stimulated echo correction (available upon request at https://mriresearch.med.ubc.ca/news-projects/myelin-water-fraction/)^47^. The analysis used 40 T_2_ relaxation delta functions logarithmically spaced from 15 to 2000 ms, 8 refocusing flip angles between 90° and 180°, and a χ^2^ regularization factor of 1.02. MWF was calculated as the fraction of the T_2_ distribution with 15 ms ≤ T_2_ < 40 ms. IET2 was calculated as the geometric mean T_2_ of components with 40 ms ≤ T_2_ < 200 ms.

### Template and atlas creation

Advanced Normalization Tools software (ANTs) was used for template creation^21^, in addition to some FSL tools^48^. The ANTs symmetric diffeomorphic normalization (SyN) transformation consistently performs similarly to, or better than, alternative techniques across a wide range of applications^21,49–51^. No quantitative maps were involved in registration, to prevent introduction of circularity bias^23^.

The template creation process is visually depicted in Supplementary Material Fig. 1. To optimize template creation, empirical testing was performed using a subset of the 100 datasets (n = 6) to reduce computational demand (processing time). Testing was evaluated by computing and comparing 3 different quantitative metrics to assess how well the images were aligned. Specifically, neighborhood cross correlation, mutual information, and global correlation similarity metrics between the 3DT1 template and each subject’s 3DT1 in template space were compared for a range of template creation parameters.

3DT1 and GRASE echo 1 images underwent N4 bias field correction to mitigate low frequency intensity nonuniformities that would affect brain extraction and registration quality^52^. GRASE echo 1 voxel intensities were squared to accentuate tissue contrast, which improved brain extractions and intra-subject registrations. This process was motivated by previous studies that manipulated image intensities for similar purposes^8^.

3DT1 and GRASE echo 1 brain extractions were guided by both structure and intensity; initialized by registration to the OASIS template and priors^53^ then refined using Atropos *n*-tissue segmentations^54^, which also provided WM, GM, and CSF segmentations for each 3DT1. The registrations used iterative rigid, affine, and SyN transformations with optimal similarity metrics for the linear (mutual information) and SyN (neighbourhood cross-correlation) stages^21^. Similarity metric calculation was constrained to within a brain and skull mask, to minimize registration errors that could arise from FOV differences between our 3DT1 images and the OASIS template.

GRASE echo 1 brain extraction masks were dilated to include CSF to prevent accidental removal of brain regions during the less accurate GRASE brain extraction. Each subject’s brain extracted GRASE image was aligned with its corresponding brain-extracted 3DT1. A rigid transformation was sufficient because neither image had evidence of geometric distortion and higher degree-of-freedom registrations did not improve the alignment. CSF regions of GRASE data (from the aforementioned generous brain extraction mask) were removed using the more accurate 3DT1 brain mask.

Input images intensities were normalized to account for differences in windowing and intensity changes from N4 correction, then rigidly aligned and averaged to create an unbiased initial template starting point. The 3DT1 template was created from unbiased co-registration of all brain-extracted 3DT1 images using the same transformations and similarity metrics as for brain extraction. The resulting 0.8 mm isotropic resolution template was created using four iterations with decreasing degrees of down sampling and smoothing. This allowed registrations to align course-resolution structure before refining fine-resolution detail. Template creation was completed in 366 h running on 21 CPU cores (2.40 GHz).

GRASE-3DT1 and 3DT1-template transformations were concatenated to warp quantitative maps from GRASE space to template space with a single interpolation step. In template space, MWF and IET2 atlases were created by calculating the voxel-wise mean, as well as median and SD, across subjects.

Binary GRASE masks were also transformed to template space and were used to exclude atlas voxels not covered by all subject GRASE datasets. This mainly affected the furthest inferior edges, where coverage was affected by variations in head size.

### Region of interest analysis

Iterative rigid, affine, and SyN transformations were used to map JHU-ICBM-DTI-81 WM labels and MNI structural regions to each subject 3DT1^55,56^. Atropos tissue segmentations were used to mask JHU labels to WM and MNI regions to exclude CSF. Then WM, GM, and CSF tissue segmentations for each 3DT1, along with the JHU and MNI ROI, were transformed to template space. These were used as inputs to the probabilistic joint label fusion framework to estimate optimal tissue segmentations and ROIs for the template^57^.

Pearson correlations were calculated between age and mean metric values extracted from voxels within 18 WM ROIs, and 1 ROI encompassing deep and cortical GM, in template space. Statistical significance was defined as *P* < 0.01. Fits to mean ROI values were used because voxel-wise analyses generally did not improve characterization of the age-MWF relationship in previous studies^12,13^. Multiple linear regression was also used to model the data with both linear and quadratic age terms. The r-values were adjusted to account for the additional model parameter, and *P*-values for age and age^2^ were provided separately to show their relative strengths. Similarly, to identify potential differences in the age-MWF relationship between males and females, multiple linear regression was also performed with an additional binarized variable for sex. Left and right ROIs were combined because age-related changes are expected to occur bilaterally^12,13^.

## Supplementary information

Supplementary Information.

Supplementary Video 1.

Supplementary Video 2.

## Data Availability

Ethics approval and subject consent were not granted for making individual subjects’ imaging data available. However, the anatomical template, quantitative atlases, and ROIs created and analysed in this study have been made openly available^16^ along with example code for their creation^17^.
