# Supplementary material for: An atlas for human brain myelin content throughout the adult life span
Source: Sci Rep. 2021 Jan 11;11:269. doi: 10.1038/s41598-020-79540-3 (PMC7801525; doi:10.1038/s41598-020-79540-3)
Supplement: Supplementary file 1 — Supplementary Information. [file 41598_2020_79540_MOESM1_ESM.pdf]

An atlas for human brain myelin content throughout the adult life span

Adam V. Dvorak[1,2]*, Taylor Swift-LaPointe[1], Irene M. Vavasour[1,3], Lisa Eunyoung Lee[4], Shawna Abel[4], Bretta Russell-Schulz[3], Carina Graf[1,2], Anika Wurl[1], Hanwen Liu[1,2], Cornelia Laule[1,2,3,5], David K.B. Li[3,4], Anthony Traboulsee[4], Roger Tam[3,6], Lara A. Boyd[7], Alex L. MacKay[1,3], Shannon H. Kolind[1,2,3,4]

[1] Physics and Astronomy, University of British Columbia, Vancouver, BC, Canada

[2] International Collaboration on Repair Discoveries (ICORD), University of British Columbia, Vancouver, BC, Canada

[3] Radiology, University of British Columbia, Vancouver, BC, Canada

[4] Medicine (Neurology), University of British Columbia, Vancouver, BC, Canada

[5] Pathology and Laboratory Medicine, University of British Columbia, Vancouver, BC, Canada

[6] Biomedical Engineering, University of British Columbia, Vancouver, BC, Canada

[7] Department of Physical Therapy, University of British Columbia, Vancouver, BC, Canada

*Corresponding Author: Adam V. Dvorak Email: adam.dvorak@ubc.ca

Room M10 Purdy Pavilion, 2221 Wesbrook Mall, Vancouver, BC, Canada V6T 2B5

Supplementary Material

Descriptions of Attached Video Files

Myelin_Atlas_Opaque.mov

Movie scrolling in the superior direction through axial slices of the mean myelin water fraction brain atlas in a Blue-Green color scale. This provides a visualization for the spatial distribution of myelin water fraction values, where high values in Green correspond to regions with high myelin content. Note that the Blue-Green color scale matches that of Figure 1 in the manuscript.

Template_Myelin_Atlas_Transparent_Superimposed.mov

Movie scrolling in the superior direction through axial slices of the anatomical brain template in grayscale, with the mean myelin water fraction brain atlas superimposed transparently in a Blue-Green color scale. This depicts the relationship between anatomical structures, shown by the underlying template, and myelin content, shown by the superimposed myelin atlas. Note that the Blue-Green color scale matches that of Figure 1 in the manuscript.

Figures and Captions

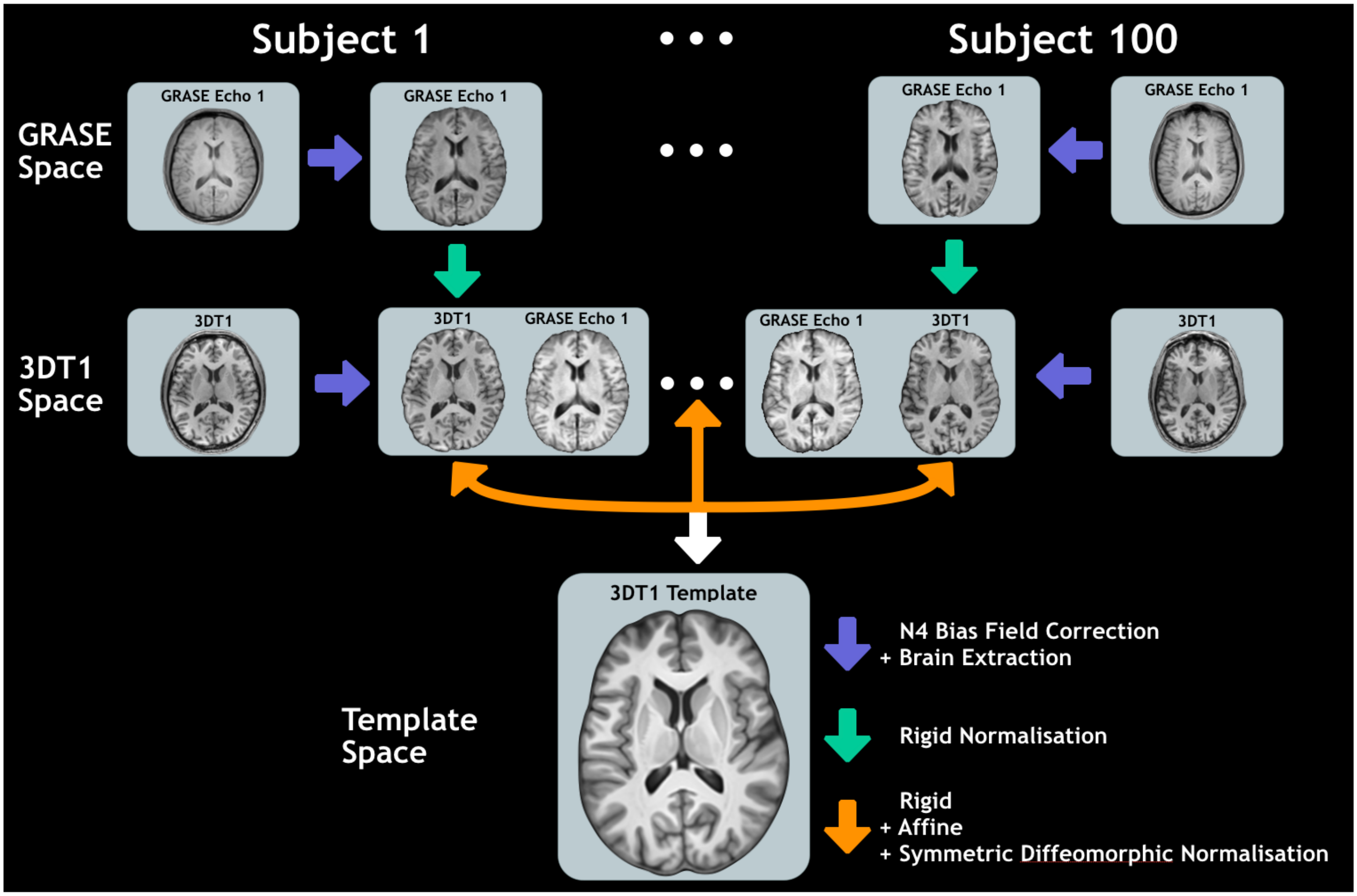

Supplementary Material Figure 1: Outline of template creation image processing and normalization for gradient and spin echo (GRASE) myelin water imaging echo 1 and T_1-weighted anatomical (3DT1) images. Before brain extraction, GRASE echo 1 voxel intensities were squared to accentuate white/gray matter contrast.
